# Creutzfeldt-Jakob Disease Presenting as Posterior Reversible Encephalopathy Syndrome

**DOI:** 10.7759/cureus.7211

**Published:** 2020-03-08

**Authors:** Jan Bittar, Parth Joshi, Justin Genova, Kevin Yeboah, Jafar Kafaie

**Affiliations:** 1 Neurology, Saint Louis University School of Medicine, St. Louis, USA; 2 Neurology, Saint Louis University Hospital, St. Louis, USA

**Keywords:** cjd, pres, prion, rapidly progressive dementia, rt quic, 1433 protein, cortical ribboning, t2 hyperintensities

## Abstract

Creutzfeldt-Jakob disease (CJD) is the most common human prion disease presenting with subacute cognitive decline. Common MRI findings for CJD include the T2 prolongation signal of the putamen and head of caudate. Diffusion-weighted MRI (DW-MRI) is considered to be the most sensitive technique for the detection of CJD-related abnormalities, especially for cortical changes. We report the case of a 77-year-old female who presented with dizziness, visual hallucination, and a rapid decline in her mental state shortly after a right knee surgery. Brain MRI with contrast showed cortical and subcortical T2 fluid-attenuated inversion recovery (FLAIR) hyperintensities in bilateral posterior temporal lobes and the left occipital lobe without an associated enhancement, suggestive of posterior reversible encephalopathy syndrome (PRES). Workup including metabolic, infectious, and vasculitic panels were all within normal limits. A few days later, she developed persistent myoclonus, and a continuous electroencephalogram (EEG) revealed multifocal epileptiform and generalized discharges, forming multifocal periodic discharges and generalized periodic discharges (GPDs). Cerebrospinal fluid (CSF) analysis was positive for 14-3-3 and elevated T-tau protein consistent with a diagnosis of sporadic Creutzfeldt-Jakob disease (sCJD). This is a rare case of CJD presenting with a brain MRI resembling PRES. CJD may have various features on MRI, and a high degree of suspicion is required to confirm the diagnosis.

## Introduction

Creutzfeldt-Jakob disease (CJD) is a subset of a larger family of fatal neurodegenerative disorders known as transmissible spongiform encephalopathies; it was first described in the 1920s by Creutzfeldt and Jakob [1]. Worldwide, the estimated incidence of CJD is 1 to 1.5 cases per million per year, and nearly 400 cases of CJD are diagnosed in the United States annually [2]. There are three forms of CJD: sporadic; familial; and acquired, which includes variant CJD. And 80-95% of the reported cases are identified to be in the sporadic form [2]. Sporadic Creutzfeldt-Jakob disease (sCJD) occurs spontaneously with a peak age of onset between 55 to 75 years old and has a poor prognosis with a 1-year mortality rate of 85-90% [2].

The Centers for Disease Control and Prevention (CDC) has outlined two criteria for probable sCJD diagnosis. This includes a neuropsychiatric disorder plus a positive real-time quaking-induced conversion (RT-QuIC) in cerebrospinal fluid (CSF) or other tissue [3]. Alternatively, rapidly progressive dementia plus at least two of the following clinical features can also be the criteria for the probable diagnosis: myoclonus, visual or cerebellar signs, pyramidal/extrapyramidal signs, or akinetic mutism in the setting of a positive ancillary test [3]. These tests include electroencephalogram (EEG) to evaluate for the presence of periodic sharp wave complexes (PSWCs), CSF for detection of 14-3-3 protein, and diffusion-weighted MRI (DW-MRI) or fluid-attenuated-inversion-recovery (FLAIR-MRI) to evaluate signal abnormalities in caudate nucleus and striatum or at least two cortical regions (temporal-parietal-occipital) in the absence of an alternative diagnosis [3]. Presently, DW-MRI is considered to be the most sensitive technique for the detection of CJD-related abnormalities, especially for cortical changes [4]. The definitive diagnosis for CJD can only be confirmed by a histological sample obtained from biopsy or autopsy [1]. 

Posterior reversible encephalopathy syndrome (PRES) is a clinic-radiological syndrome characterized by reversible changes occurring within the central nervous system that can be viewed by MRI or CT of the brain [5]. PRES classically appears as diffuse cortical, subcortical, and deep lesions within the posterior regions of the brain, and it is estimated that most cases involve either the parietal or occipital lobes [5]. CJD should be considered in the differential diagnosis of PRES if the lesions affect the basal ganglia and cerebral cortex [5]. 

Here we report an atypical presentation of CJD with brain MRI findings typically associated with PRES.

## Case presentation

**Figure 1:**
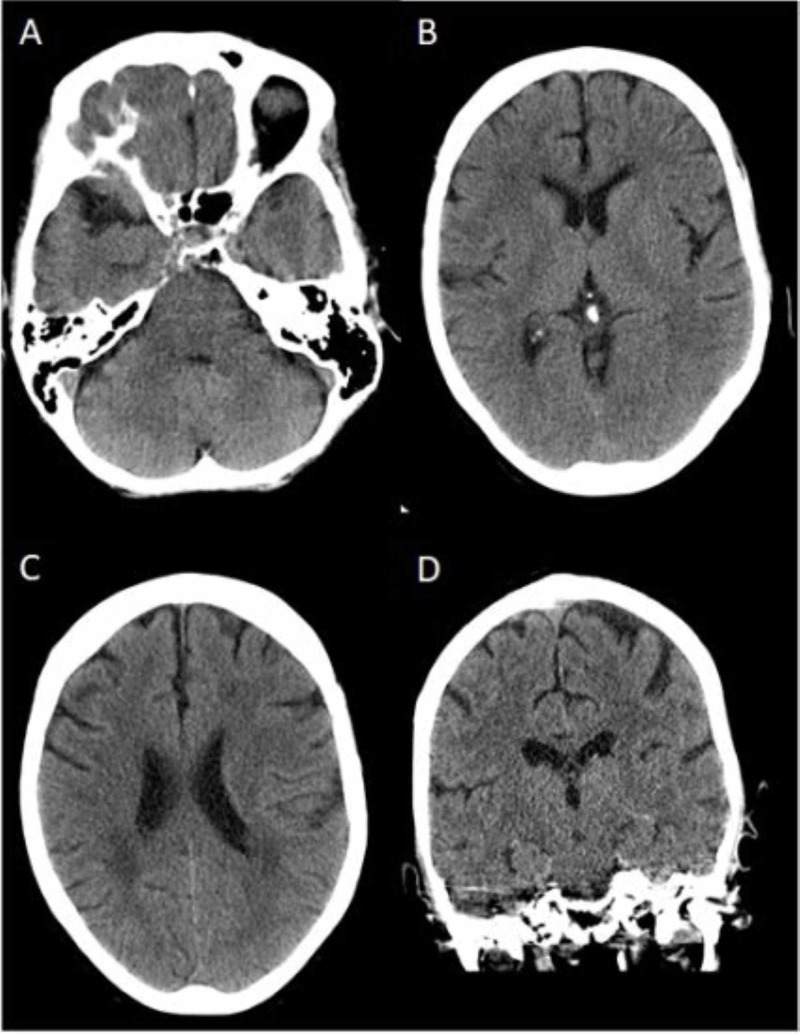
CT of the head without intravenous contrast A,B,C: axial view; D: coronal view No acute intracranial process is seen CT: computed tomography

**Figure 2:**
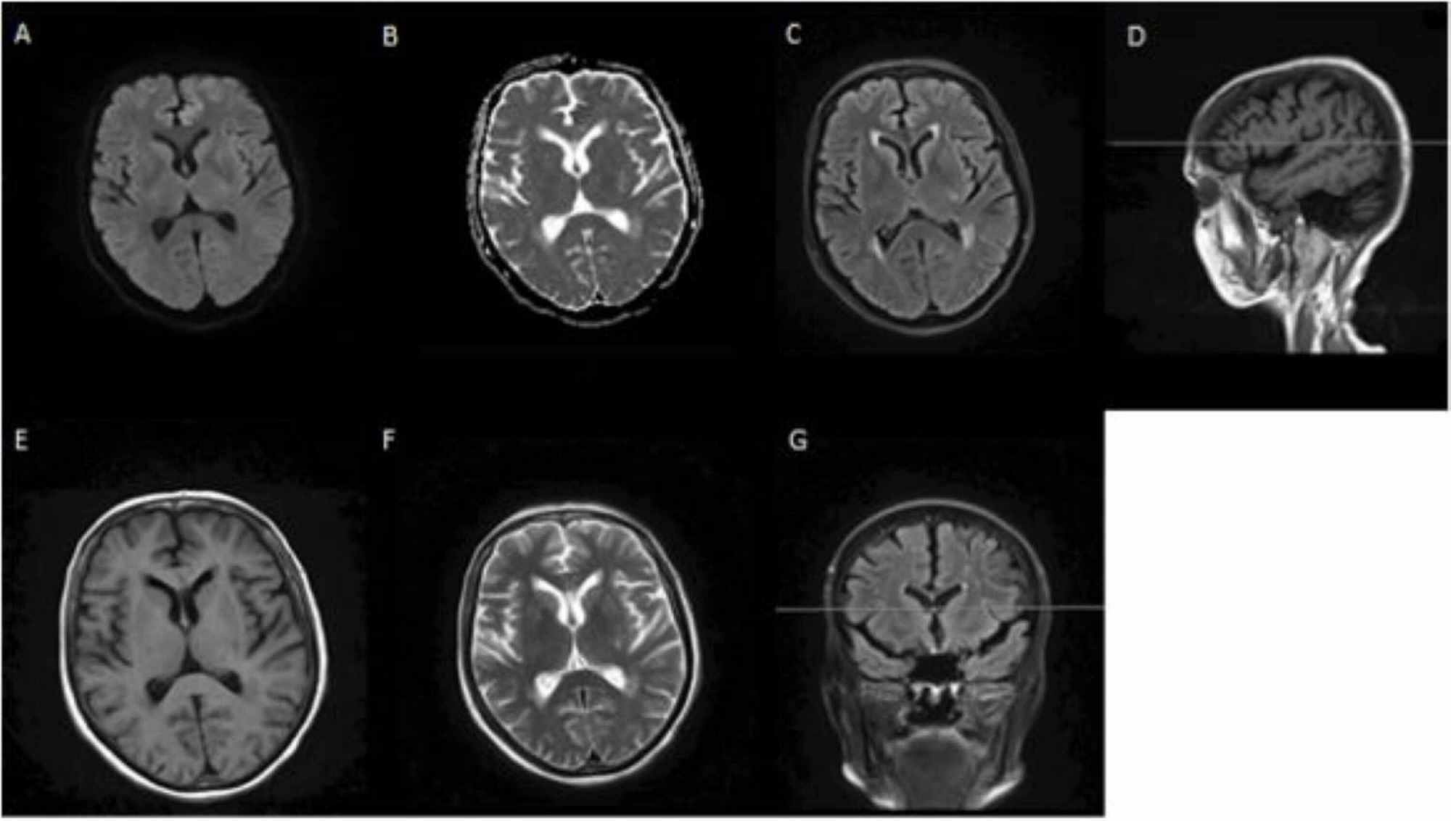
MRI of the brain without intravenous contrast A: axial DWI; B: axial ADC; C: axial T2 FLAIR; D: sagittal T1 FLAIR; E: axial T1 FLAIR; F: axial T2; G: coronal T2 FLAIR Mild age-appropriate generalized cerebral atrophy. No acute infarction or foci restricted diffusion are identified in DWI and ADC maps. Nonspecific and chronic supratentorial white matter changes are seen MRI: magnetic resonance imaging; DWI: diffusion-weighted imaging; ADC: apparent diffusion coefficient; FLAIR: fluid-attenuated-inversion-recovery

A 77-year-old female with a history of chronic alcohol abuse presented with dizziness and confusion one month after a right knee replacement for degenerative joint disease. She described her dizziness as “lightheadedness” that was not associated with any specific maneuver or changes in position. Prior to undergoing knee surgery, she had been healthy, independent, and abstinent from alcohol. With the onset of the confusion, her family had found her to be “jittery” with sleep disturbances and difficulty concentrating. On examination, she was found to have impaired orientation to time and slow thought processing. Her Mini-Mental State Exam (MMSE) was 21/30, consistent with mild cognitive impairment, and the remainder of her systemic exam was unremarkable. A noncontrast CT of the head was negative for an acute intracranial process, and a follow-up MRI of the brain without contrast was unrevealing for underlying abnormality (Figures 1-2). Given her prior history of chronic alcohol intake, supplementation with thiamine and folic acid was initiated.

**Figure 3:**
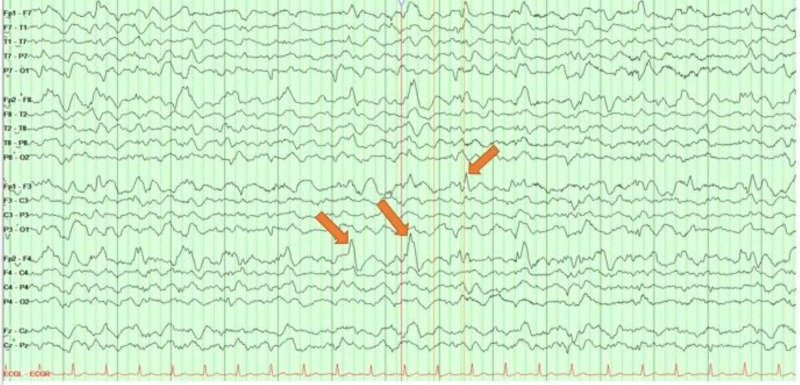
A longitudinal bipolar montage (double banana) routine electroencephalogram Generalized background slowing and high amplitude sharply contoured delta or theta activity over the bilateral frontopolar region with shifting predominance more visible over the right (Fp2 > Fp1) arrows. There is a brief interval of right frontopolar faster alpha frequencies

**Figure 4:**
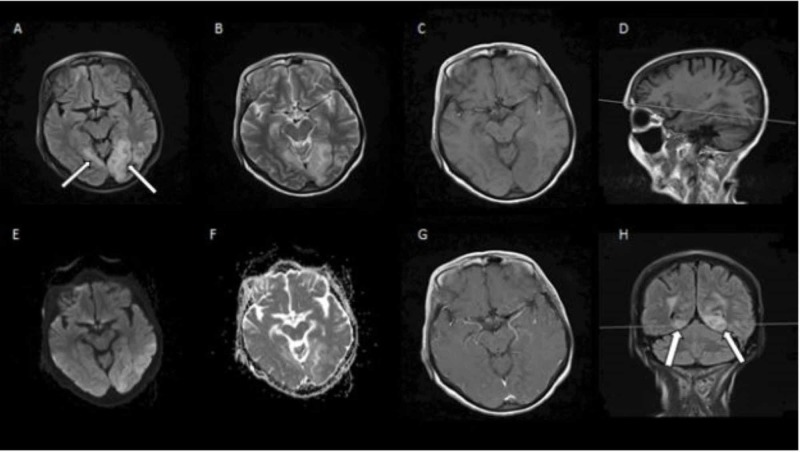
MRI of the brain with and without intravenous contrast A: axial T2 FLAIR; B: axial T2; C: axial T1 FLAIR; D: sagittal T1 FLAIR; E: axial DWI; F: axial T2 ADC; G: axial T1 with contrast; H: coronal T2 FLAIR Cortical and subcortical T2 FLAIR hyperintensity involving the bilateral posterior temporal lobes and the left occipital lobe (arrows), representing posterior reversible encephalopathy syndrome. There is no evidence of acute infarction. No enhancing lesions are identified MRI: magnetic resonance imaging; FLAIR: fluid-attenuated-inversion-recovery; DWI: diffusion-weighted imaging; ADC: apparent diffusion coefficient

One week later, the patient started to complain of visual hallucinations. An EEG was obtained to rule out seizures and showed bifrontal discharges more visible over the right hemisphere with right frontal faster frequencies suggestive of an increased tendency toward seizures. Generalized background slowing was also observed, suggestive of moderate to severe encephalopathy (Figure 3). A repeat brain MRI with contrast showed cortical and subcortical T2 FLAIR hyperintensities in bilateral posterior temporal lobes and the left occipital lobe without associated enhancement (Figure 4). The MRI demonstrated features suggestive of PRES. Workup including metabolic, infectious, and vasculitic panels in serum and CSF were all unremarkable with the exception of elevated antinuclear antibodies (ANA) at 1:80. A trial of high-dose methylprednisolone (1 g for 5 days) was administered due to concern for central nervous system vasculitis or autoimmune-mediated process. The patient showed no clinical improvement after completing a course of steroid therapy.

**Figure 5:**
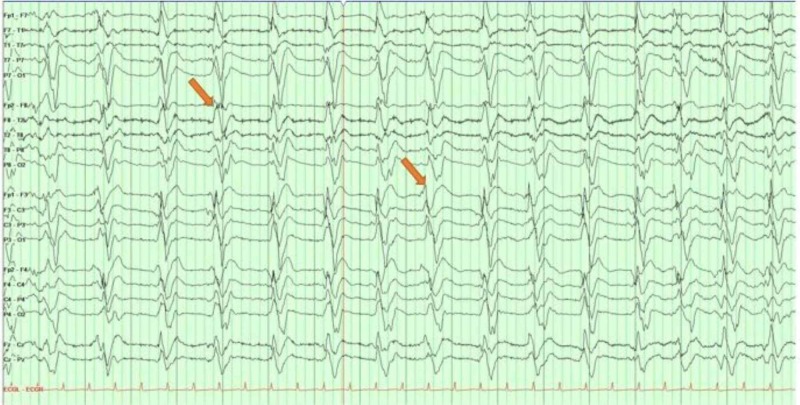
A longitudinal bipolar montage (double banana) continuous electroencephalogram Generalized periodic discharges (GPDs) at the frequency of 1 discharge every 0.5 to 1 second (arrows)

**Figure 6:**
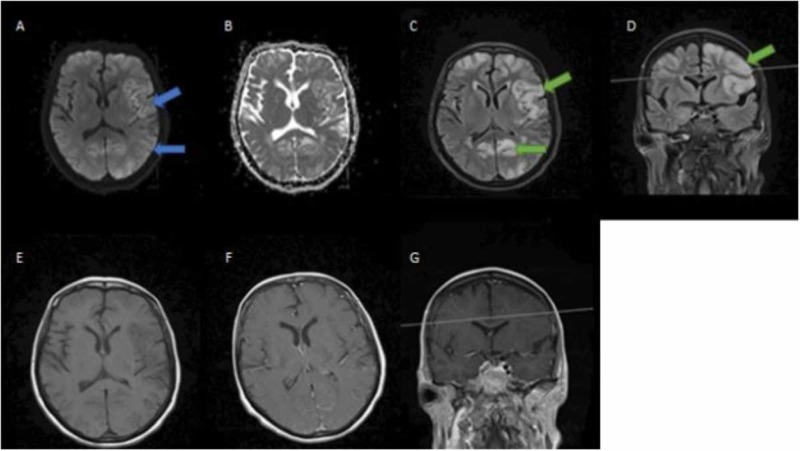
MRI of the brain with and without intravenous contrast A: axial DWI; B: axial ADC; C: axial T2 FLAIR; D: coronal T2 FLAIR; E: axial T1 FLAIR; F: axial T1 with contrast; G: coronal T1 with contrast There is an interval increase in the cortical and subcortical T2 FLAIR hyperintensities (green arrows), now involving bilateral cerebral cortex diffusely with no associated enhancement. There is new diffusion restriction in frontoparietal, temporal, insular, and occipital cortex on DWI sequences representing cortical ribboning (blue arrows), which is suggestive of CJD pattern MRI: magnetic resonance imaging; DWI: diffusion-weighted imaging; ADC: apparent diffusion coefficient; FLAIR: fluid-attenuated-inversion-recovery; CJD: Creutzfeldt-Jakob Disease

Nineteen days after the admission, her condition significantly deteriorated with frequent episodes of agitation and jerky myoclonic movements involving her orofacial and proximal upper limbs. Continuous electroencephalogram (cEEG) revealed multifocal epileptiform and generalized discharges, forming multifocal periodic discharges (MfPDs), and generalized periodic discharges (GPDs) at 0.5-1 Hz suggestive of CJD (Figure 5). A repeat brain MRI with contrast showed diffuse cortical and subcortical T2 FLAIR hyperintensities without enhancement. Additionally, new diffusion restrictions in frontoparietal, temporal, insular, and occipital cortex were noted consistent with cortical ribboning characteristic of CJD (Figure 6). A repeat lumbar puncture was performed, and CSF was sent for 14-3-3 and t-tau protein analysis.

Twenty-five days after the admission, she became increasingly somnolent and less responsive and was subsequently intubated. She was then transferred to the neurointensive care unit (NICU) where she remained intubated and sedated for the duration of the hospital course. She continued to decompensate and the family requested to withdraw care. Previous CSF results had returned positive for 14-3-3 and an elevated t-tau protein consistent with the diagnosis of sCJD. Autopsy and brain biopsy were not performed.

## Discussion

Our case demonstrates an atypical presentation of CJD with features of PRES on MRI. Of all available neuroimaging modalities, MRI is the most helpful method in the diagnosis of CJD [6]. Recent studies show that diffusion-weighted imaging (DWI) and apparent diffusion coefficient (ADC) sequences have a sensitivity of 92-96% and a specificity of 93-94% for CJD [2]. Common locations for subcortical involvement include hyperintensities within the basal ganglia and thalamus, with a special sign called the “pulvinar sign” characterized by bilateral FLAIR hyperintensities involving the pulvinar thalamic nuclei [2]. This sign has been reported in at least 75% of variant CJD cases and has been rarely seen in other forms of CJD [2]. High cortical signal intensity on DWI or FLAIR is also well reported in sCJD [4]. The most common MRI findings of cortical involvement include high-intensity signals in the insula, cingulum, superior frontal cortex, and cortical areas near the midline [4]. It is unclear why CJD has a predilection for these structures; however, associations between signal intensity changes and specific clinical signs have been supported [4,7]. Interestingly, DWI is superior to FLAIR in the detection of these cortical high-signal intensities [4].

While there has been an increase in the use of MRI for diagnosing CJD, atypical findings can be noted in the early clinical stages of sCJD [8]. Interestingly, our MRI revealed cortical and subcortical T2 FLAIR hyperintensities involving bilateral posterior temporal lobes and the left occipital lobe. The posterior region is a classic location for abnormalities to be seen in PRES and approximately 98% of patients exhibit this distribution [5]. It is believed that rapid blood pressure rise in PRES impairs cerebral blood flow autoregulation and disturbs the blood-brain barrier (BBB), resulting in cerebral hyperperfusion and vasogenic edema [9]. The sympathetic nervous system causes cerebral vasoconstriction to protect the brain against severely elevated blood pressures [9]. However, the posterior circulation of the brain has a less robust sympathetic innervation, which may explain the characteristic MRI findings associated with PRES [9]. Additionally, severe hypertension may overload the sympathetic autoregulatory response in the middle cerebral arteries (MCA), which gives rise to the medullary arteries supplying the deep white matter and temporal poles [9]. The medullary arteries have also been shown to have minimal sympathetic innervation, making these regions vulnerable to the extreme pressures underlying PRES [10]. These mechanisms are proposed to explain the typical white matter findings in PRES.

Common risk factors for the development of PRES include arterial hypertension, eclampsia or preeclampsia, chemotherapy regimens, organ transplants that require immunosuppressant medications, severe infection, chronic renal failure, and autoimmune diseases [5]. In our case these risk factors were absent; however, the typical pattern seen in PRES on MRI was demonstrated. This distribution has rarely been identified in CJD with only two other reported instances in the literature. One case report describes a woman who presented with rapid forgetfulness over the span of a week with initial MRI findings significant for bilateral-occipital-cortical enhanced signal intensity in FLAIR and DWI sequences suggestive of PRES. A few weeks later, she developed limb rigidity, action tremor, severe apraxia, gait instability, and akinetic mutism. CSF results were positive for 14-3-3 protein [11]. A second case report described a patient with initial MRI findings showing increased signal-intensity zones in cortical gray and subcortical white matter, predominantly in the right parietal-occipital area without associated diffusion restriction, leading to an initial diagnosis of PRES. A repeat MRI showed symmetrical lesions in the basal ganglia, and this along with a typical constellation of symptoms led to the consideration of CJD as the most likely diagnosis, which was later confirmed by brain biopsy. [8].

In rare cases, PRES can present with edema involving the cerebellum, basal ganglia, brain stem, and more anterior structures such as the frontal lobe [5]. Due to overlapping findings between PRES and CJD, it may be feasible to encourage the use of alternative methods to establish the diagnosis of CJD. RT-QuIC is a new CSF test shown to have a sensitivity of 85-87% and specificity of 99-100% for sCJD [12]. This new technique makes use of recombinant prion protein (rPrP) that induces aggregates when CSF fluid containing the disease-associated form, PrPSc, is added [13]. Thioflavin T (ThT) is then introduced to bind to aggregated PrPSc and cause monitorable ThT emissions in real time [13]. While this new method has a higher sensitivity and specificity than either 14-3-3 or tau protein, it is not without shortcomings; for example, if the CSF has total protein concentrations of >1.0 g/L and an elevated white cell count, it can lead to false-positive results [14].

This work has been presented as an abstract at the Neurocritical Care Society (NCS) 16th Annual Meeting (https://link.springer.com/article/10.1007%2Fs12028-018-0606-9).

## Conclusions

CJD is a subset of a larger family of fatal neurodegenerative disorders known as transmissible spongiform encephalopathies. We reported an atypical presentation of CJD with brain MRI features resembling PRES. CJD may have variable MRI features and a high degree of suspicion is required to establish the diagnosis.
